# Policy proposals by children during the COVID-19 pandemic through the global child rights dialogues in Japan, Sweden and Tanzania

**DOI:** 10.1136/bmjpo-2026-004674

**Published:** 2026-05-25

**Authors:** Hajime Takeuchi, Malale Tungu, Mazen Baroudi

**Affiliations:** 1 Faculty of Social Welfare, Bukkyo University, Kyoto, Japan; 2 Department of Developmental Studies, Muhimbili University of Health and Allied Sciences, Dar es Salaam, Tanzania, United Republic of; 3 Department of Epidemiology and Global Health, Umeå University, Umeå, Sweden

**Keywords:** Child Health, COVID-19, Health Policy, Social work

## Abstract

**Background:**

In the early stages of the COVID-19 pandemic, children were not the primary carriers of the infection. However, during the pandemic, children’s everyday lives were significantly restricted, making it difficult to gather with one another, play and learn in person.

**Objectives:**

To clarify the status of their rights under 10 selected articles of the Convention on the Rights of the Child (CRC) during the pandemic and to propose policies that enable children to enjoy their rights through the Global Child Rights Dialogue (GCRD) process.

**Methods:**

Between June 2022 and January 2024, 293 children in Japan, Sweden and Tanzania participated in 46 groups. Using a ‘Research with Children’ approach, they discussed the status of each CRC article and suggested policy proposals to advance the rights set out in the CRC. Text mining was used to analyse frequently occurring words. Their problem statements and policy proposals were further examined and discussed. All proposals, as outlined in each article, were aggregated and analysed in a single co-occurrence network for each country.

**Results:**

The proposals reflected each country’s unique circumstances while also including universal demands that transcend national borders. For example, in Japan, school rules disregarded children even before COVID-19, and in Tanzania, the exclusion of children from public transportation due to lower fares had been raised as a problem. Common issues across the three countries included violations of children’s rights, particularly restrictions on opportunities to play and learn together. Many of their proposals, such as eliminating gender altogether, were insightful beyond what adults would have considered.

**Conclusion:**

Through the GCRD, children clarified the violations of their rights under the COVID-19 pandemic and articulated policy demands to address them. Our study highlights the value of actively engaging children in research, which has implications for public policy and academia globally.

**WHAT IS ALREADY KNOWN ON THIS TOPIC**

- Global Child is an international group of researchers that organised the Global Child Rights Dialogue (GCRD) in 2018, which developed platforms for setting standards and monitoring children’s rights in many settings.
- We know that children’s rights were violated during the COVID-19 pandemic in many different ways and that young people had views about these violations.

**WHAT THIS STUDY ADDS**

- The children were able to recognise violations of their rights under each article during the COVID-19 pandemic and articulate the policy proposals needed to advance those rights.

**HOW THIS STUDY MIGHT AFFECT RESEARCH, PRACTICE OR POLICY**

- Conducting research with active engagement of children, even led by children, is a highly valuable endeavour that should become standard practice globally. Giving children the opportunities to discuss their rights through meaningful channels allows them to share their perspectives and policy recommendations, which should in turn influence policy and planning.

## Introduction

 In the early stages of the COVID-19 pandemic, children were not the primary carriers of the infection.^1^ However, schools were closed entirely for 3 months in Japan. Following the declaration of a state of emergency in urban areas in April 2020, school closures were extended and many schools would not reopen until June 2020.^2^ Schools were also closed for 3 months in Tanzania.^3^ In Sweden, schools for children under 16 were only temporarily closed when local COVID-19 case numbers were high. Regardless of these differences, school closures, staggered attendance and shortened school days were implemented.^4^

Combined with playground closures, this significantly restricted children’s everyday lives, preventing them from gathering with one another, playing and learning together.^5^

Article 12, paragraph 1 of the Convention on the Rights of the Child (CRC) states, ‘States Parties shall assure to the child who is capable of forming his or her own views the right to express those views freely in all matters affecting the child, the views of the child being given due weight in accordance with the age and maturity of the child’. This proclaims the right of children to express their own views.

Global Child (GC), an international group of child development and child rights researchers, advocates and experts based in Canada and globally, launched an initiative called the Global Child Rights Dialogue (GCRD) in 2019, in which 52 organisations in 35 countries around the world proposed the standards for children to determine whether they are enjoying the rights set out in the CRC.^6^ The International Society for Social Pediatrics and Child Health, of which the lead author is a member, requested that we participate in the GCRD, which led to the implementation of the GCRD in Japan. This project aimed to clarify how children’s rights were violated during the pandemic through the GCRD conducted by children in three countries and to enable children to propose political solutions at the local and global levels. The GCRD is a discussion process in which children in each country share their views and propose policies on child rights in each CRC Article.

### Current situation of the convention on the rights of the child

Japan became the 158th of 196 countries to ratify the CRC in April 1994. The prohibition of corporal punishment was codified in law through amendments to the Child Welfare Act and other relevant laws, and it came into effect in April 2020. The UN Committee on the Rights of the Child emphasised the need to allocate sufficient public budgets to reduce the high child poverty rate, to enact comprehensive anti-discrimination laws, to recognise joint custody of children and to ensure that no child is tried in adult criminal courts. Because the age of criminal responsibility was lowered from 16 to 14 in 2000.^7^ Government surveys have revealed that many children are taking on household and family care roles during the pandemic and that some of this care by young carers constitutes child labour.^8^

Sweden was the first country in the world to legally prohibit corporal punishment of children in 1979. It was one of the original signatories to the CRC, which came into force in 1990. It established a Children’s Ombudsman in 1993 and, in 2020,^9^ became the first country to incorporate the CRC into domestic law. However, challenges remain in implementing these principles in practice. Sahlberg *et al* noted that children’s medical care must explicitly take their rights into account.^10^ Furthermore, the 2023 Concluding Observations of the UN Committee on the Rights of the Child recommend strengthening children’s participation in decision-making at the local level and ensuring the equitable provision of public education for all children from socioeconomically disadvantaged families, including refugees.^11^

Tanzania ratified the CRC in 1991 but has not yet enacted legislation prohibiting corporal punishment. In its second periodic report in 2006, Tanzania was recommended to enact the Children’s Act.^12^ This recommendation was followed in 2009 with the enactment of the Children’s Act, formally titled the *Law of the Child*.^13^ In 2011, the Human Rights Council of the UN pointed out several areas for improvement including concerns about the minimum marriage age of 15 for girls, the discrimination against children in socioeconomical difficulties and street children, problems with the functioning of juvenile councils, the lack of a provision prohibiting corporal punishment, the harmful custom of female genital mutilation and educational law forcing pregnant girls to drop out of school.^14^

### Research with children

This project employs a ‘research with children’ approach. In Europe, a paradigm shift is occurring in research methods involving children. Christensen and Prout proposed a new research methodology for children that extends beyond the traditional view of children as subjects in research and society, positioning them as participants and co-researchers.^15^ Looking back at the history of research approaches, Kellett argues for a shift from traditional experimental research on children or social research about children to participatory research with children, or research conducted by children themselves.^16^ Christensen *et al* published Research with Children: Perspectives and Practices in 2000 and released the third edition in 2017.^17^ This book clarifies the methodology of research practices with children. In the introduction chapter, they concluded that ‘Only through listening and hearing what children say and paying attention to the way in which they communicate with us will progress be made toward conducting research with children’.

Furthermore, the CRC transformed children from ‘objects of protection (passive beings)’ to ‘subjects of rights (active beings)’ who possess their own rights.

When designing research on children, we researchers must prioritise the protection of children’s rights and practice and establish research ‘with children’ or ‘by children’.

### Japan, Sweden and Tanzania

The selection of Japan, Sweden and Tanzania was based on the first author’s research connections (opportunity sampling). Basic socioeconomic data for these three countries, including children, sourced from the World Bank and OECD Data, are shown in table 1.

**Table 1:** Indicators and data from each country

Country	Population 2023*		Fertility rate* 2023	GDP per capita* 2023	Gini index* (0–100)	Foreign population† 2024 (%)
	Total	% aged 0–14				
Japan	124 517 000	12	1.2	33 836	32.3 (2020)	3 400 000 (2.7%)‡
Sweden	10 537 000	17	1.4	54 950	29.3 (2023)	2 200 000 (20.5%)§
Tanzania	66 618 000	43	4.6	1224	40.5 (2018)	N.D.

* World Bank Data.

† OECD Data.

‡ Foreign population.

§ Foreign-born population.

## Methods

The GCRD groups were selected through purposive and snowball sampling. The GCRD implemented period was 19 months from June 2022 to January 2024. In Japan, 38 GCRD sessions were conducted with 104 children in 19 groups (two sessions per group) at 13 locations nationwide from June 2022 to October 2023, spanning from Ishinomaki City, Miyagi Prefecture, in the east to Takamatsu City, Kagawa Prefecture, in the west. In Sweden, 20 GCRD sessions were conducted with 44 children across 10 groups (two sessions per group) at a single location in Umeå City from February 2023 to January 2024. In Tanzania, 17 GCRD sessions were conducted with 145 children in 17 groups (one session per group) at secondary schools, both ordinary and advanced levels, in Dar es Salaam and its surrounding areas over 1 month in July 2023.

### Patient and public involvement

The child-friendly CRC articles used in our GCRD project were created by 13–14-year-old girls. GCRD efforts in each country were made possible with the cooperation of local staff and participants’ parents. We plan not only to publish the results of this research in papers, but also to share the children’s policy proposals in booklets that are easy for children to understand.

### The GCRD process was carried out using the following steps

1. Each researcher used a combination of telephone, written communication and in-person visits to explain the outline of the initiative to the staff of a facility (school, leisure activity organisation or other facilities) and obtain their approval.
2. The staff of a facility facilitated the recruitment of children. Each group consisted of 3–10 children aged 9 to 18, and, if possible, multiple groups were recruited at a single facility.
3. Once the GCRD implementation date was confirmed, an explanation of the GCRD and consent forms for families and children was sent to facility staff in advance.
4. On the day of the implementation, facility staff were asked to set up the venue, and researchers worked with them to prepare for the implementation.
5. When the GCRD was implemented, only the supporting facility staff and researchers were present. No family members or facility staff were allowed to be present during data collection.
6. The GCRD process is as follows:
  - The facilitator prepared a child-friendly version of one CRC article; arranged desks and chairs to facilitate discussion; and prepared documents, writing tools and drinks and snacks for the children.
  - Once all the children gathered, the facilitator introduced himself or herself and explained how the GCRD would proceed.
  - While enjoying drinks and snacks, the children created name tags and introduced themselves to each other and filled out consent forms for participation and audio recording.
  - The discussion of the articles (GCRD) lasted approximately 1 hour.
  - The facilitator provided hints on how to proceed but was careful not to lead the discussion and respected the children’s independent discussion.
  - The contents of the GCRD for each article and the policy proposals that emerged from it were presented by the children’s group.
  - After the presentations, everyone was given a certificate of completion, marking the end of the project.

We selected 10 articles from the CRC for the GCRD. Those were; the four principles of CRC, that is, non-discrimination (Article 2); the best interests of the child (Article 3); the right to life, survival, development (Article 6); and respect for the views of the child (Article 12), in addition to six articles including the right of children with disabilities (Article 23); the right to health and health services (Article 24); the right to social security (Article 26); the right to a decent standard of living (Article 27); the right to education (Articles 28 and 29); and the right to leisure, play and engage in cultural activities (Article 31). Articles 28 and 29 were treated as a single category of education.

**Figure 1:**
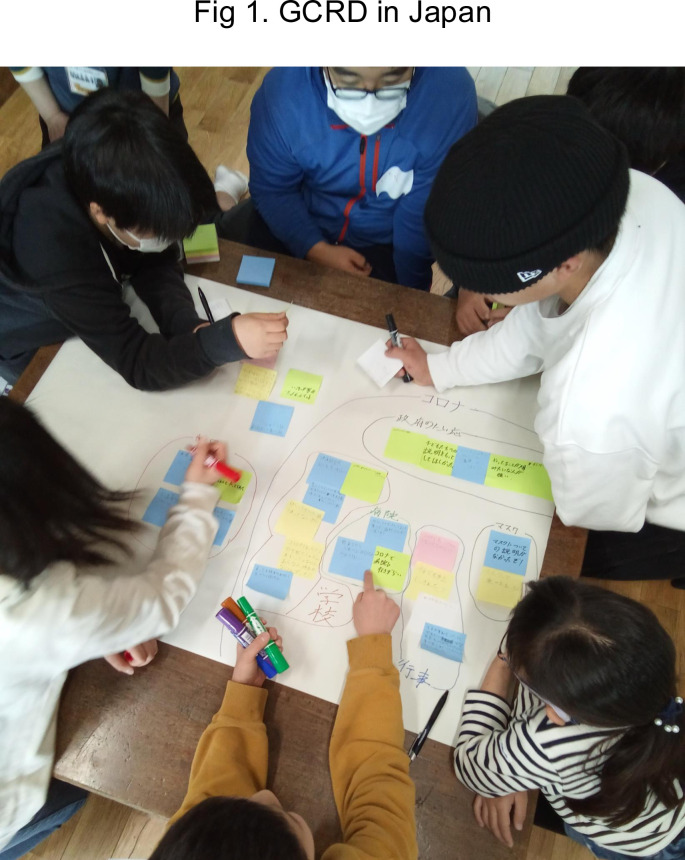
GCRD in Japan. GCRD, Global Child Rights Dialogue.

In Japan and Sweden, each group discussed two articles. In Tanzania, each group discussed one article. Figure 1 shows an example of how GCRD was conducted.

### The KH coder analysis

All GCRD recorded by the facilitators were transcribed verbatim in their original language (Japanese, Swedish and Swahili). The researchers reviewed the transcripts and extracted/summarised the problem statements and policy proposals for each dialogue in English. The problem statements and policy proposals were edited to ensure clarity, including replacing all pronouns with the intended nouns. These summaries were then analysed quantitatively for each of the three countries using text mining with the KH Coder.^18^ First, to standardise the expressions used in each country, terms such as ‘corona virus’, ‘corona (time)’, ‘the corona infection’, ‘corona infection’, ‘corona’, ‘Corona’, ‘corona pandemic’, ‘the pandemic’ and ‘the COVID-19 pandemic’ were all referred to as ‘COVID-19’. ‘In corona time’, ‘during the pandemic’ and ‘during the COVID-19 pandemic’ were all referred to as ‘during COVID-19’. ‘Student’ and ‘students’ were standardised to ‘child’ and ‘children’. When selecting words in the KH Coder, the following were excluded: proper noun; foreign words; unclassifiable characters and symbols; frequently used verbs used in a variety of contexts (be, do, go, have, hold, give, take, send, become, get and use); pronouns (I, my, your, it, his, her, we and they and it); adjectives (other, many, high, more, less and due); and adverbs (much, too and also).

After preprocessing, quantitative text analysis was performed with a minimum occurrence threshold of 10, and the top 60 co-occurrence relationships were selected for Japan’s GCRD. In this case, six groups emerged. Therefore, to ensure that the number of co-occurrence groups in each country was six, the top settings for co-occurrence relationships were adjusted to six group occurrences. When the top thresholds were set to the top 45 co-occurrences for Sweden and 30 for Tanzania, mutual comparisons were facilitated, that is, resulted in six group occurrences, and these thresholds were adopted.

To aid interpretation of the co-occurrence network, each country’s co-occurrence network is accompanied by sentences that briefly outline the messages within that group. These sentences were reviewed, revised and agreed on by the three co-researchers.

### Distinctive policy proposals based on KH coder analysis

Based on the dialogue analysed using co-occurrence networks, we identified country-specific policy proposals and universal proposals common to all countries. We then linked these proposals to the political and socioeconomic contexts of each country in the discussion section.

### Ethical considerations

In conducting this study, approval was obtained from the ethical review committee at Bukkyo University for ‘research involving human subjects’ in Japan (approval number 2–21-33-B). In Sweden, approval was obtained from the Swedish Ethical Review Board (Dnr 2022–04241-01). In Tanzania, approval was obtained from the MUHAS Research and Ethics Committee (ref. No. DA. 2821 298/01. C/).

## Results

Online supplemental table S1 presents the number of articles discussed at the GCRD in each country, the number of children participating, and their gender and age.

The children who participated were 104 from Japan (55 boys, 48 girls and 1 who did not wish to specify their gender), 44 from Sweden (27 boys and 17 girls) and 154 from Tanzania (79 boys and 75 girls).

The children’s ages ranged from 9 to 17 years; however, in Tanzania, the GCRD was held in secondary schools, so no children under 13 were recruited. In Japan, there were three 18-year-old participants, including older siblings and high school senior groups. In Japan, there were three 18-year-old participants, including older siblings of other participants and a group of high school seniors aged 17 and 18.

### Quantitative text analysis using the KH coder

The total number of extracted words and the number of times each part of speech was extracted are shown in online supplemental table S2. The top frequently appearing words (more than 10 occurrences) are shown in online supplemental table S3. Common frequent words across countries were the strong association between ‘child’ and ‘not’.

**Figure 2:**
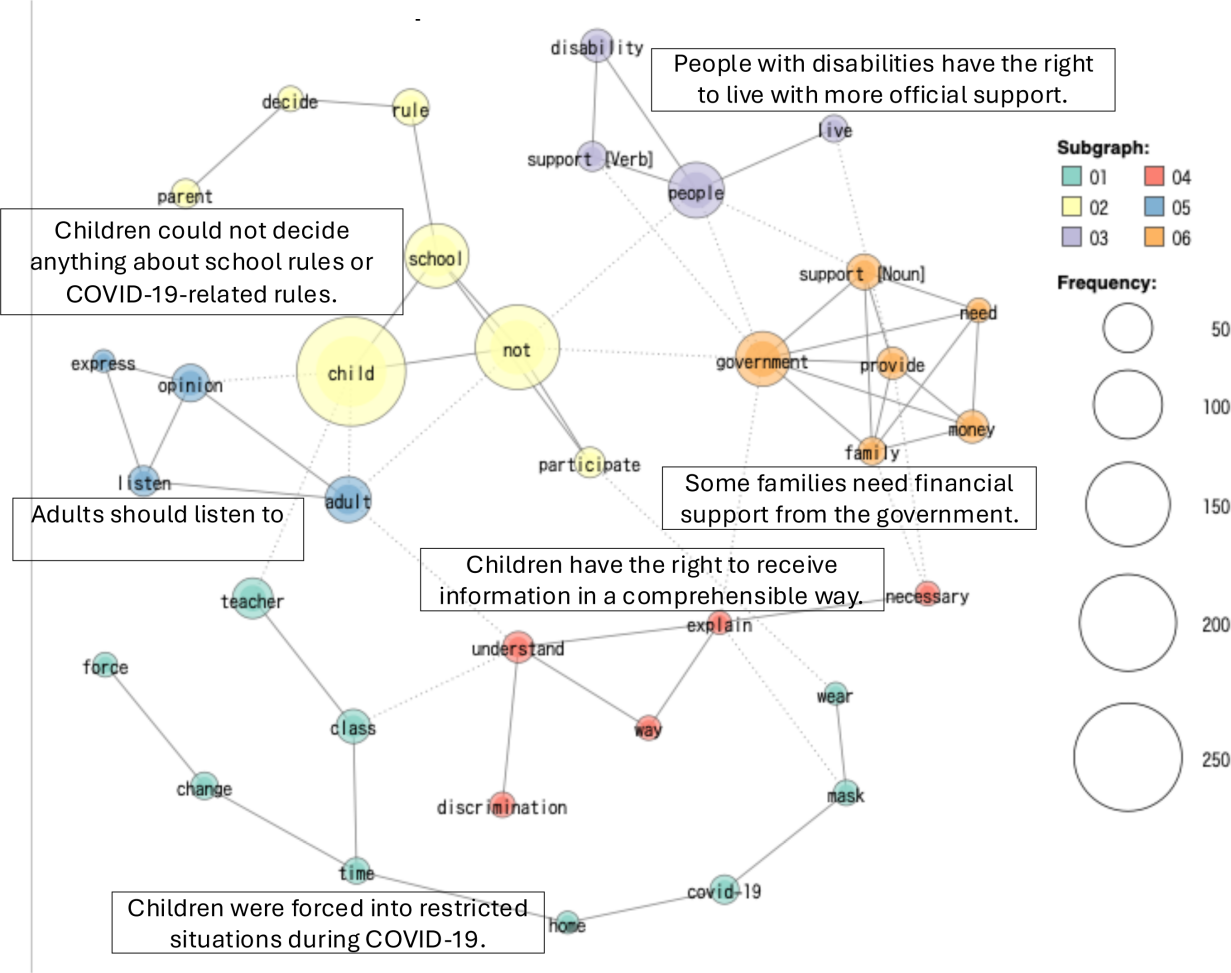
Japan co-occurrence network.

The Co-occurrence Network results for each country are shown in figures2^4^. The analysis identified six groups (co-occurrence networks) by country-level GCRD.

**Figure 3:**
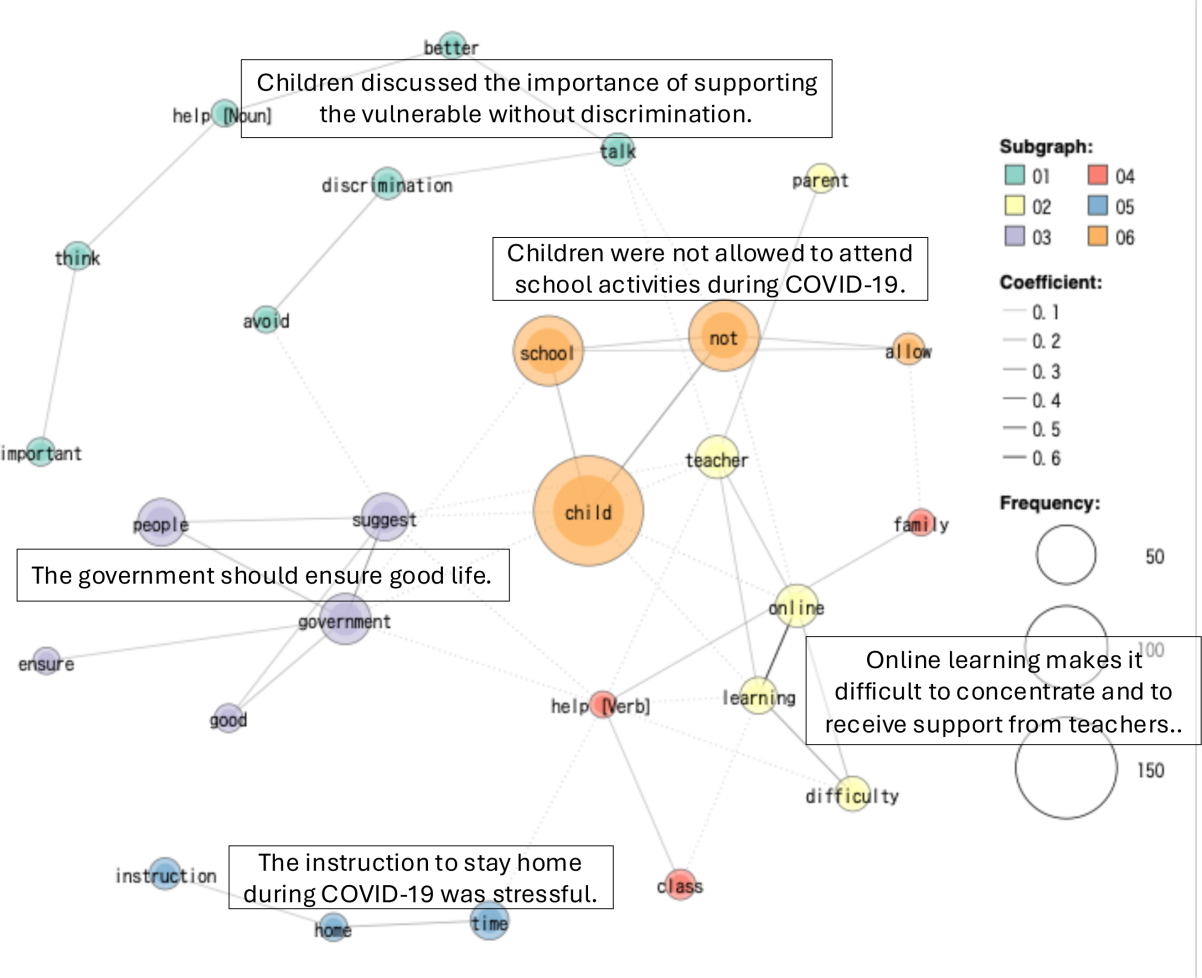
Sweden co-occurrence network.

**Figure 4:**
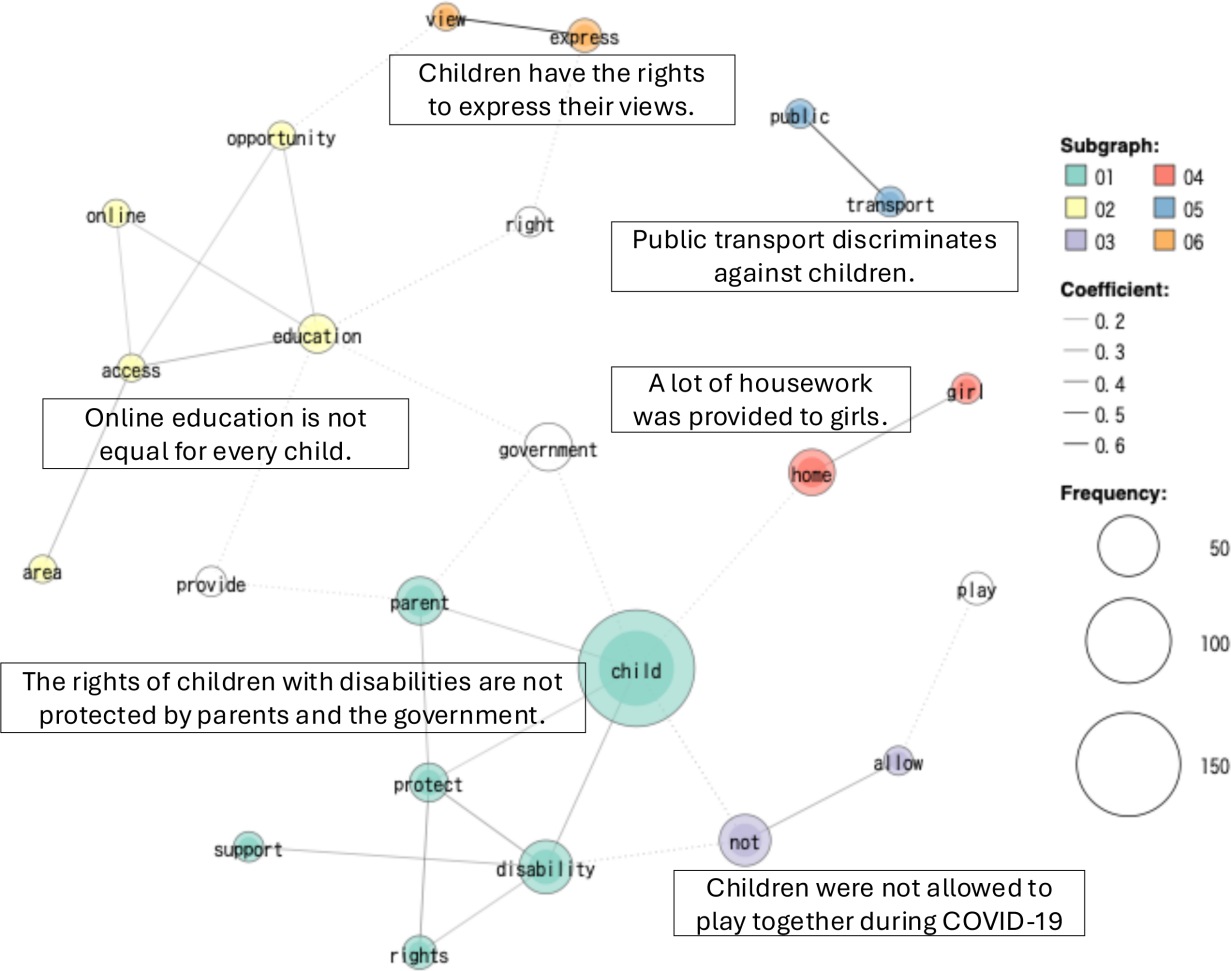
Tanzania co-occurrence network.

These co-occurrence networks include 83% of the top-frequency words in Japan, 76% in Sweden and 82% in Tanzania.

### Children’s explanation for the violations of their rights and their proposed policy proposals

#### Japan

The GCRD in Japan was expressed through six co-occurrences. Participants included children from diverse backgrounds, including those with family problems, not attending school, with disabilities and living in child welfare institutions. In the largest co-occurrence, children expressed a stifling sense of being bound by school rules that predated the pandemic and demanded that their opinions be considered in school decision-making, especially regarding pandemic-related rules, such as the necessity of wearing masks and eating lunch in silence, separated by panels. They also called for gender diversity, including the abolition of single-sex education and even the abolition of gender itself. They also raised issues and proposed measures to address the root causes of discrimination, such as eliminating discriminatory practices and preventing adults from conveying discriminatory beliefs to children. They also made policy proposals that questioned the nature of society, such as realising a society in which failure is tolerated, and all children can feel happy to be alive. They also called for government support for young carers and for the implementation of mental health checks alongside physical health examinations in schools. They also called for the development of painless injections and treatments, and for the provision of accessible spaces for children to gather and eat outside the home, particularly for children without a suitable home environment.

#### Sweden

The co-occurrence network analysis of the Swedish dialogues reveals six co-occurrences. The most prominent co-occurring terms (child, not and school) highlight how children’s everyday lives were restructured by pandemic measures, particularly restrictions on after-school social interaction and changes in learning environments. Children consistently emphasised fairness and non-discrimination, stressing that all children should be treated equally regardless of place of birth, language background or disability. Difficulties associated with online learning emerged as a key concern, especially for foreign-born children and those struggling with Swedish, who faced barriers in accessing timely support from teachers. At the same time, children proposed concrete solutions, such as increasing teacher availability, providing special teachers for language support and offering psychological support through school nurses for those experiencing fear or stress. The analysis also shows that children valued being listened to, suggesting that their inclusion in school decision-making processes, including voting on school measures, would better safeguard their rights. While many children reported stress, reduced well-being and a perceived decline in their standard of living due to strict instructions to stay at home, some also acknowledged that government and school management of the pandemic was good.

#### Tanzania

Six co-occurrences were also identified in Tanzania with child, disability and not being the most frequently appearing in the GCRD. During the pandemic, children experienced loneliness because they were not permitted to go outside to play together. Children with disabilities were not supported well due to the social distancing and the school closures. Many girls were involved in household chores. As a result, girls missed out on fun activities. Furthermore, some girls faced problems with early pregnancy. Children’s rights to access health services and education were not protected during COVID-19, and barriers prevented children from attending church. The problem that children were excluded from public transport violated children’s rights. This was because children paid less than adults and were not allowed to board buses. Children proposed as follows. It was important to have a special room or offices with privacy for them to report on gender-based violence and related issues. The government should provide public transport for all, regardless of fare rank. Children should have the right to play without the burden of child labour. Online sessions should be accessible to everyone, including those in rural areas.

## Discussion

The policy proposals concluded by the children’s discussions in the GCRD have been quantitatively analysed using the KH Coder method in the Results chapter. In this Discussion chapter, we aim to clarify the ‘Pearls of wisdom’ that the children demonstrated through the GCRD, which surpass the thinking of adults, and the problem-solving proposals that could only be presented from children’s perspectives, while focusing on proposals based on the specific circumstances of each country and proposals for the protection of universal rights, and discuss them deeply with references.

### Violations of children’s rights and policy proposals

Across all three countries, a common theme of the pandemic was the violation of children’s rights, particularly restrictions on opportunities to play and learn. Children in the three countries also reported that the severity of COVID-19’s impact on children varied depending on various discrimination grounds, including disability, residential location or immigration status. Another common theme across the three countries is the importance of authorities actively listening to children’s concerns about their rights.^19^ Children emphasised the need for their voices to be heard in decision-making processes that affect their lives, highlighting that meaningful participation is essential for ensuring their rights are respected and protected. A significant difference between the Swedish children’s GCRD and those in the other two countries was that the children stated that there was a system in place to communicate their rights in schools. Although the situation is insufficient, the system allows children to make policy proposals^9^ (Box 1).

Box 1 **Common violations and policy proposals of children’s rights**

- Children were restricted in opportunities to play and learn during the pandemic.
- Severity of the impact on children varied across various situations of discrimination.
- The authorities should actively listen to children’s concerns regarding their rights.
- Children’s voices should be heard in decision-making processes affecting their lives.

#### Violations of children’s rights and policy proposals in Japan

Japanese children’s policy proposals included some groundbreaking proposals not often offered by adults. For example, they not only questioned how children were taught discriminatory ideas but also suggested eliminating the concept of discrimination and gender in the first place. They also called for ‘accepting every child as they are’ and ‘creating a society where children feel that their lives are valued and that they have a future to look forward to, even if they currently struggle with self-confidence’. Furthermore, children questioned school rules that fail to sufficiently respect children as individuals often stipulating detailed regulations on children’s belongings, hairstyles and clothing. The process of deciding these rules lacks student involvement, and although many schools have student councils, negotiations with school authorities are often merely formalities.^20^

This situation stems from the fact that the Basic Act on Education had not previously referred to children’s rights or to CRC. The new Basic Act on Children’s Policy, enacted in 2023, is the first to explicitly state that policies would be pursued in accordance with the spirit of the CRC. The task of bridging the gap between the existing Basic Act on Education and the new law remains to be done.

The proposal from children to include ‘mental health checkups’ in school health checkups should be taken seriously and introduced. School absenteeism is increasing during and after the pandemic, and suicide is the leading cause of death among teenagers. Especially, over half of those aged 15–19-year-olds die by suicide.^21^ This situation is not limited to children. The number of teachers taking leave due to mental illness is on the rise.^22^ According to statistics from the Ministry of Education, Culture, Sports, Science and Technology, this has increased by approximately 1.5-fold over the past 10 years, reaching 1 in 130 teachers. The average number of students per class in elementary and junior high schools is 27.2 and 32.0, respectively, nearly 1.5 times Sweden’s numbers of 20.1 and 21.8, respectively.^23^ Therefore, teachers face greater demands as they handle more students.

#### Violations of children’s rights and policy proposals in Sweden

Sweden incorporated the CRC into domestic law in 2020, requiring the state to consider children’s opinions in decisions affecting their welfare and support services. The law enshrines the ‘best interests of the child’ as a core principle, mandating that policymakers, agencies and courts prioritise children’s well-being in all relevant decisions.^24^ Unlike children in Japan and Tanzania, who rarely expressed positive opinions about government policies, children in Sweden praised the government’s response to the COVID-19 pandemic. However, a report indicated violations of children’s rights during the pandemic, including their rights to survival, life and health, as well as to education, protection and care.^25^ Swedish children reported that, despite schools largely remaining open, their daily lives were still affected, especially in play, learning and social interaction.^26^ Children described feeling fear, stress and anxiety about contracting the virus or losing loved ones, reflecting global trends in childhood mental health during COVID-19.^27^,^29^ Limited school health staff, particularly in rural areas, further reduced access to mental health support.^30^ Inequities in education were evident: children with disabilities, special educational needs or limited technology faced greater challenges, and students from disadvantaged families were disproportionately affected.^31 32^ Children emphasised that communication during the pandemic was insufficiently child-focused. They called for mechanisms that allow them to actively participate in decisions affecting their lives, rather than merely receiving information.^33 34^ These insights highlight the need for equitable access to education, strengthened school-based mental health support and participatory communication structures to uphold children’s rights during crises.

As shown above, the government should prioritise expanding school-based mental health services and provide targeted support to socioeconomically marginalised groups. Sweden appears to be developing mechanisms that enable children to participate in decision-making more than the other two countries. However, it needs further development to establish children’s participatory mechanisms within schools or local governance structures.^26^

#### Violations of children’s rights and policy proposals in Tanzania

Tanzania generally protects children’s rights through the Law of the Child Act (2009).^35 36^ In accordance with the CRC, this law guarantees the right to a name, nationality, education and protection from abuse. The government has strengthened child protection efforts through initiatives such as establishing child centres. Specifically, 30 centres have been established in major cities to enhance the protection of vulnerable children, particularly street children.^37^ However, significant challenges remain, including high rates of corporal punishment of children at home and school and sexual violence in the home and community. This also highlights the widespread forced domestic labour of especially girls in many households and calls for government intervention in families and parents to address this worsening violation of children’s gender-neutral rights to play and learn.^38^,^40^ Children have called on the government to protect girls from sexual harassment by their communities and families and questioned the heavy burden placed on girls in household activities. The GCRD also revealed that COVID-19 has made it difficult for children to use public transportation. This is due to a policy known as ‘level seating’. ‘Level seat’ was a strict rule during COVID-19, which required passengers on public transportation (city buses) to sit rather than stand, thereby preventing overcrowding and congestion.^41^ Originally part of the Road Traffic Act, it has become a significant focus of attention during the pandemic. As a result, children paying lower fares have been excluded from public transportation. The children called for fair access to buses.

Through this GCRD, it has become clear that the situation of girls in particular is even more serious. The children demanded that the government should educate adults to prevent girls from becoming victims of sexual abuse. This is an earnest proposal. There was a group that raised concerns about the significant role of religion and how children’s right to attend mosques and churches was taken away during the COVID-19 pandemic.

Table 2 summarises frequently occurring or particularly important messages on child-rights violations through GCRD, along with policy proposals, across three countries.

**Table 2:** Individual violations and policy proposals of children’s rights

Japan	The largest opinion was that the school rules did not reflect the children’s voices.
	The children suggested eliminating the concept of discrimination and gender.
	One policy recommendation was to create a society in which failure is tolerated.
	The children called for government support for young carers.
	The mental health checks should be added to school health examinations.
Sweden	The children struggling with Swedish had difficulty accessing the online support.
	Children from disadvantaged families were disproportionately affected.
	The children proposed more teachers and school nurses for psychological support.
	The children called for mechanisms in which they can participate in decisions.
	Some acknowledged that the government management of the pandemic was good.
Tanzania	Children with disabilities were not supported well during the pandemic.
	Girls were forced to do household chores and missed out on play and learn.
	The children called on the government to protect girls from sexual harassment.
	The children called for fair access to buses, even though the fares were lower.
	Online sessions should be accessible to everyone, including those in rural areas.

### Strengths and limitations

The strength of this study is embodying the concept of ‘research with children’. By clearly explaining what GCRD is and which CRC article to discuss, children were able to proactively analyse the current state of their rights and make policy recommendations for improvement. This was possible despite weaknesses such as younger age or developmental disabilities. Children themselves took the lead in the GCRD project, discussed infringements of their rights due to COVID-19 and proposed policies to address these issues. Therefore, this study is groundbreaking in that it clearly demonstrates the necessity of empowering and even enabling children to be active participants in advancing the articles of the CRC. However, there were some challenges in applying this approach; it was sometimes difficult to continue discussions when the group consisted of children of a younger age or when participants had developmental disabilities. In such cases, we intervened by selecting the articles under discussion to be more specific ones, such as Article 31, which concerns the right to play and cultural activities, or by having a facilitator orient the discussion. In addition, children at some GCRDs in Japan struggled to engage in discussion because they were not familiar with one another before the session. Because the GCRD was implemented in junior and high schools, those under 12 years old were excluded. There were restrictions on the age range in Tanzania. Finally, although the GCRD in Japan was conducted across a wide area of the country, the results in the other two countries showed limited geographic variation, limiting our ability to generalise our findings to the whole of Sweden or Tanzania.

## Conclusion

Through the GCRD, 10 articles on children’s rights enshrined in the CRC were discussed, and children shared their experiences of their rights during the pandemic and articulated policy demands to advance them. The proposals reflected each country’s unique circumstances while also including universal demands that transcend national borders.

Children are not merely beings to be protected or expected to obey adults. Through GCRD, they could articulate their opinions and contribute policy proposals to their country. Many of their proposals were insightful beyond what adults would have considered. As adults, we need to listen attentively to children’s voices and take them seriously in decision-making. Our findings confirm that engaging children in research represents an endeavour of significant academic value.

It has also confirmed the importance of our society listening to children’s opinions, whether regarding future pandemics, existing conflicts or climate crises.

## Supplementary material

10.1136/bmjpo-2026-004674 online supplemental table 1

10.1136/bmjpo-2026-004674 online supplemental table 2

10.1136/bmjpo-2026-004674 online supplemental table 3

## Data Availability

Data are available upon reasonable request.
