# Supplementary material for: Policy proposals by children during the COVID-19 pandemic through the global child rights dialogues in Japan, Sweden and Tanzania
Source: BMJ Paediatr Open. 2026 May 25;10(1):e004674. doi: 10.1136/bmjpo-2026-004674 (PMC13202095; doi:10.1136/bmjpo-2026-004674)
Supplement: online supplemental table 1 [file bmjpo-10-1-s001.pdf]

Supplemental Table S1 Number of each article and participating children

Number of GCRD for each article	Article 2	Article 3	Article 6	Article 12	Article 23	Article 24	Article 26	Article 27	Article 28+29	Article 31
Japan	4	3	4	4	4	4	4	3	4	4
Sweden	3	1	2	1	3	1	2	1	3	3
Tanzania	3	2	2	2	1	1	1	2	2	1
**Number of Children by age and gender**	**9 years old**	**10 years old**	**11 years old**	**12 years old**	**13 years old**	**14 years old**	**15 years old**	**16 years old**	**17years old**	**18 years old**
Japan	3	13	16	17	13	7	21	4	7	3
Total: 104	Girls / Boys / Others = 55 / 48 / 1									
Sweden	0	5	4	10	8	6	6	4	1	0
Total: 44	Girls / Boys / Others = 27 / 17 / 0									
Tanzania	0	0	0	0	14	23	38	44	35	0
Total: 154	Girls / Boys / Others = 79 / 75 / 0									
