# Supplementary material for: Policy proposals by children during the COVID-19 pandemic through the global child rights dialogues in Japan, Sweden and Tanzania
Source: BMJ Paediatr Open. 2026 May 25;10(1):e004674. doi: 10.1136/bmjpo-2026-004674 (PMC13202095; doi:10.1136/bmjpo-2026-004674)
Supplement: online supplemental table 2 [file bmjpo-10-1-s002.docx]

| Supplemental Table S2 Co-occurrence network analysis | | | | | |
| --- | --- | --- | --- | --- | --- |
| Country | Extracted total words | Noun | Verb | Adjective | Adverb |
| Japan | 2,990 | 1,608 | 806 | 336 | 240 |
| Sweden | 2,234 | 1,198 | 559 | 322 | 155 |
| Tanzania | 1,495 | 913 | 365 | 149 | 68 |
