# Supplementary material for: Policy proposals by children during the COVID-19 pandemic through the global child rights dialogues in Japan, Sweden and Tanzania
Source: BMJ Paediatr Open. 2026 May 25;10(1):e004674. doi: 10.1136/bmjpo-2026-004674 (PMC13202095; doi:10.1136/bmjpo-2026-004674)
Supplement: online supplemental table 3 [file bmjpo-10-1-s003.docx]

| Supplemental Table S3 Top frequent words (Grey words: not appearing in the co-occurrence network) | | | | | | | | |
| --- | --- | --- | --- | --- | --- | --- | --- | --- |
| Japan | | | Sweden | | | Tanzania | | |
| Word | Part of Speech | Frequency | Word | Part of Speech | Frequency | Word | Part of Speech | Frequency |
| child | Noun | 256 | child | Noun | 177 | child | Noun | 190 |
| not | Adverb | 152 | not | Adverb | 73 | Disability | Noun | 39 |
| school | Noun | 87 | school | Noun | 71 | not | Adverb | 36 |
| people | Noun | 65 | government | Noun | 37 | government | Noun | 31 |
| government | Noun | 63 | people | Noun | 32 | parent | Noun | 31 |
| adult | Noun | 44 | suggest | Verb | 32 | home | Noun | 29 |
| teacher | Noun | 34 | teacher | Noun | 26 | education | Noun | 20 |
| opinion | Noun | 29 | online | Adjective | 26 | protect | Verb | 20 |
| rule | Noun | 27 | time | Noun | 21 | school | Noun | 18 |
| support | Noun | 26 | learning | Noun | 19 | health | Noun | 15 |
| class | Noun | 24 | COVID-19 | Noun | 18 | rights | Noun | 15 |
| disability | Noun | 22 | difficulty | Noun | 17 | express | Verb | 15 |
| money | Noun | 22 | Talk | Verb | 15 | play | Verb | 14 |
| provide | Verb | 21 | discrimination | Noun | 14 | COVID-19 | Noun | 12 |
| understand | Verb | 20 | instruction | Noun | 14 | girl | Noun | 12 |
| create | Verb | 19 | allow | Verb | 14 | right | Noun | 12 |
| listen | Verb | 19 | need | Verb | 14 | transport | Noun | 12 |
| participate | Verb | 19 | class | Noun | 13 | provide | Verb | 12 |
| support | Verb | 18 | news | Noun | 12 | support | Verb | 12 |
| COVID-19 | Noun | 17 | parent | Noun | 12 | public | Adjective | 11 |
| parent | Noun | 17 | health | Noun | 11 | allow | Verb | 11 |
| family | Noun | 16 | home | Noun | 11 | access | Noun | 10 |
| change | Verb | 15 | good | Adjective | 11 | area | Noun | 10 |
| live | Verb | 15 | important | Adjective | 11 | food | Noun | 10 |
| trouble | Noun | 15 | think | Verb | 11 | opportunity | Noun | 10 |
| time | Noun | 14 | family | Noun | 10 | view | Noun | 10 |
| play | Verb | 14 | help | Noun | 10 | online | Adjective | 10 |
| discrimination | Noun | 13 | information | Noun | 10 |  |  |  |
| mask | Noun | 13 | opinion | Noun | 10 |  |  |  |
| decide | Verb | 13 | better | Adjective | 10 |  |  |  |
| event | Noun | 13 | avoid | Verb | 10 |  |  |  |
| bully | Verb | 13 | ensure | Verb | 10 |  |  |  |
| way | Noun | 12 | express | Verb | 10 |  |  |  |
| necessary | Adjective | 12 | follow | Verb | 10 |  |  |  |
| force | Verb | 12 | help | Verb | 10 |  |  |  |
| need | Verb | 12 | try | Verb | 10 |  |  |  |
| explain | Verb | 11 | use | Verb | 10 |  |  |  |
| increase | Verb | 11 |  |  |  |  |  |  |
| home | Noun | 10 |  |  |  |  |  |  |
| express | Verb | 10 |  |  |  |  |  |  |
| wear | Verb | 10 |  |  |  |  |  |  |
| environment | Noun | 10 |  |  |  |  |  |  |
