# Supplementary material for: Policy proposals by children during the COVID-19 pandemic through the global child rights dialogues in Japan, Sweden and Tanzania
Source: BMJ Paediatr Open. 2026 May 25;10(1):e004674. doi: 10.1136/bmjpo-2026-004674 (PMC13202095; doi:10.1136/bmjpo-2026-004674)
Supplement: Supplementary file 4 [file bmjpo-2026-004674.reviewer_comments.pdf]

PEER REVIEW HISTORY

BMJ Paediatrics Open publishes all reviews undertaken for accepted manuscripts. Reviewers are asked to complete a checklist review form and are provided with free text boxes to elaborate on their assessment. These free text comments are reproduced below.

ARTICLE DETAILS

**TITLE (PROVISIONAL)**	Policy Proposals by Children during the COVID-19 pandemic through the Global Child Rights Dialogues in Japan, Sweden, and Tanzania
**AUTHORS**	Takeuchi, Hajime<br>Tungu, Malale<br>Baroudi, Mazen

VERSION 1 – REVIEW

**REVIEWER NAME**	Zwi, Karen
**REVIEWER AFFILIATION**	University of New South Wales - Randwick Campus, School of Women's and Children's Health
**REVIEWER CONFLICT OF INTEREST**	No Conflict of Interest
**AI DISCLOSURE**	No, I have not used AI
**DATE REVIEW RETURNED**	10-Mar-2026

**GENERAL COMMENTS**	This piece is a valuable contribution to the literature regarding the rights and opinions of children in relation to COVID pandemic policy recommendations. It is very impressive that the authors managed to obtain the views and recommendation of almost 300 children from 3 very different countries, all of which experienced unique issues. The article is generally well written and succinct. I think it could be enhanced by further discussion of what policy demands and suggestions the children actually made and more of an exploration of how the children's different circumstance in each country affected their perceptions. Overall this is an outstanding effort to include the voices of children in our policy recommendations and I commend the authors for this work.<br><br>PLEASE SEE DETAILED SUGGESTIONS BELOW<br>Abstract<br>'Common issues' are presented but please add the unique issues.<br>'Proposals were insightful' - please add further detail in the abstract (and the text) about what these proposals actually were.<br>Key messages<br>What is already known: This section requires a rewrite for clarification eg 'Global Child, an internation group of [what is it] promotes and supports children/CRC [what the organisation aims to do and achieves].<br>This sentence does not make sense to me: 'The children could make platforms to measure child-related issues through GCRD' – please clarify.<br>What this study adds<br>Please add the specifics of what the children proposed and recommended.<br>Introduction<br>Well written and informative.

	Minor editing:<br>P4 L15 incomplete sentence<br>L17 restricted<br>L33: standards for children<br>P5 L6-9 There is no need to detail the book title etc – this is in the references. Rather summarise the key concepts the authors propose<br>Section 2.1<br>Please explain why only girls were involved in creating the CRC articles<br>Results<br>P8 L22 is not clear – perhaps split into2 sentences. What happened if children turned 18 during the study?<br>Tables could be far more engaging and clearly presented eg Table 1 list the ages (perhaps in categories in a column on left).<br>Table 3 insert dividing lines between countries.<br>Consider a Table of Policy Recommendations as they seem to get lost throughout this article.<br>3.2.3 Tanzania<br>The 'level seats' issue should be explained the first time it is presented.<br>'Dedicated desks' may not be clear for an international audience – does this refer to call-in centres/drop in centres?<br>Discussion<br>Overall well written and interesting. Please add more on the children's policy recommendations and possible next steps. Also please reflect more on how the unique experiences of children from different countries comes through in their perceptions. This is a very interesting aspect that is not explored.<br>My suggestion is that '4.1 Current situation of the CRC' should be moved to the Introduction and properly integrated there. It disturbs the flow of the reader where it currently sits.<br>'Strengths and limitations': please elaborate on any difficulties encountered with children with disabilities and what adaptations were made for them.<br>P15 L13 It is not clear what is meant by the 'age restrictions in Tanzania"<br><br>Conclusions<br>As before, what policy recommendations were the most significant that we need to listen to? How did children's unique country experiences during the pandemic affect their perception and recommendations? Why is this important ie it may be that we face future pandemics.

**REVIEWER NAME**	Iwamoto, Azusa
**REVIEWER AFFILIATION**	Japan Institute for Health Security,, Division of Public Relation and Communications
**REVIEWER CONFLICT OF INTEREST**	No Conflict of Interest
**AI DISCLOSURE**	No, I have not used AI
**DATE REVIEW RETURNED**	24-Mar-2026

**GENERAL COMMENTS**	**Overall impression:**<br>This article introduces a novel and important global topic for readers: the Global Child Rights Dialogue (GCRD) and 'Research with Children' approach. Moreover, the authors adopt GCRD in three countries in Africa, Asia and Europe. Therefore, the contents

	are worth publishing although it needs several major revisions. Especially, the whole structure of this manuscript seems not well organized (each content of **Background**, **Results** and **Discussion** is mixed up together) so changing the configuration of structure is recommended.<br><br>**Comments for each section:**<br>1. 'Policy Recommendations' in **Title** is a keyword of this article. However, it is difficult to find the policy recommendations in **Result** section although there are several sentences on **Figure 2,3,4**. Which parts are the policy recommendation? (one table will be needed).<br>**Key Messages:**<br>2. The whole writing style should be considered carefully again (Please refer to the published articles of BMJPO and following their writing structure. All sentences are written in the same way). Especially, **What this study adds**' part is written in distinctive styles, so it is difficult what they mean.<br>**Introduction**<br>3. As 1-2, the basic information on children in Japan, Sweden and Tanzania should be explained (e.g., geographic characteristics, total fertility rate, school enrollment rate, rate of migrants…). In addition, the reason these three countries were selected should be explained.<br>Methods<br>4. P5L19-20: study period'<br>What does it mean? (by the end of all interviews??<br>5. P524-25: 'spanning from Ishinomaki City… in the west'<br>This part cannot be understood for foreigners because of too detail. So, this part maybe no need).<br>6. P5L36: '**Patient and Public Involvement**':<br>What does it mean??<br>7. P5L51: approval'<br>How was it obtained? Orally?<br>8. P6L10-31:<br>The process can be described as one paragraph. In the process **ⅳ)**, the role of children should be explained, such as 'one volunteer for presentation'.<br>9. P6L512: transcribed verbatim'<br>Did you use any recording tool? If so, it should be explained.<br>10. P7L21-30:<br>In this part, it is difficult to understand why six group occurrences were adopted in Sweden and Tanzania after six groups emerged in Japan's GCRD. Please explain the process in more detail.<br>11. P7L37:<br>Are the meanings/definitions of 'policy proposals', 'policy demand' and 'policy recommendation' the same or not? Please use these key words with a clear purpose.<br>12. P7L33: sentences that briefly outline the messages within that group'<br>What is the relationship between this phrase and the 'policy recommendations?<br>**Results**<br>13. **Table1**:<br>It is difficult to see tables because 1) the characters are too small, and 2) each item and numbers are not coordinated so we cannot understand the meaning of this table. Please remake it.<br>14. **Table2 and Table3**:<br>The character of each item is too small.<br>15. P10L19-20: 'The following…'<br>This sentence is no need.

	16. P11L40: Level seats :<br>These words appear suddenly so it needs some explanation or should be expressed with “”. The explanation appeared on P14L44 for the first time.<br>**Discussion**<br>17. At the first paragraph of Discussion, 1) strengths of this study, and 2) statement of main (two or three) discission points should be described.<br>18. P11-12: 4.1 **Current situation of the CRC**<br>This paragraph is not discussion but basic information so it is better to move to **Introduction** section.<br>19. P12-13: **4.2.1-4.2.3**<br>These discussions for three countries seem to be nearly results. It is not clear how the authors extract these contents<br>from **Result** section. I apology repeating but where are policy recommendations which we find easily? And are they the same as policy proposals/policy demands/sentences that briefly outline the messages within that group’ ? These parts make the reader confused.<br>20. P14L54: Missing **4.3**<br>As one section (maybe 4.3?) in **Discussion**, it is better to put the reflection of comparison for policy recommendation among three countries, in a summary style. Such multi-countries evaluation will show the worth conducting the study in three countries.<br>21. P14L55: **Strengths**<br>To what extent this study is brand-new, innovative and unique should be underlined first. It means that it is important to mention how much the research with children- approach and GCRD are popular in the world when policy recommendations are formulated on CRC.<br>**Acknowledgements**<br>22. It is written in heart-warning style but not formal. No need ‘Thank you, XX.’

VERSION 1 – AUTHOR RESPONSE

Reviewer: 1

Comments to the Author

This piece is a valuable contribution to the literature regarding the rights and opinions of children in relation to COVID pandemic policy recommendations. It is very impressive that the authors managed to obtain the views and recommendation of almost 300 children from 3 very different countries, all of which experienced unique issues. The article is generally well written and succinct. I think it could be enhanced by further discussion of what policy demands and suggestions the children actually made and more of an exploration of how the children’s different circumstance in each country affected their perceptions. Overall this is an outstanding effort to include the voices of children in our policy recommendations and I commend the authors for this work.

Thank you very much for the comments.

PLEASE SEE DETAILED SUGGESTIONS BELOW

Abstract

‘Common issues’ are presented but please add the unique issues.

‘Proposals were insightful’ - please add further detail in the abstract (and the text) about what these proposals actually were.

→We added a sentence: ‘For example, in Japan, school rules disregarded children even before COVID-19, and in Tanzania, the exclusion of children from public transportation due to lower fares had been raised as a problem.’ And we add one significant example, ‘such as eliminating gender altogether’.

Key messages

What is already known: This section requires a rewrite for clarification eg ‘Global Child, an internation group of [what is it] promotes and supports children/CRC [what the organisation aims to do and achieves].

This sentence does not make sense to me: ‘The children could make platforms to measure child-related issues through GCRD’ – please clarify.

What this study adds

Please add the specifics of what the children proposed and recommended.

→Within the constraints of the text, we rewrote it as follows:

Global Child: It is an international group of researchers that organised the Global Child Rights Dialogue (GCR.D) in 2018 and developed platforms.

It is difficult to add an example because of the limitation on the number of sentences of Key Messages.

→Within the constraints of the text, I rewrote ‘What this study adds’ as follows:

Violations of children's rights: Through the same GCRD process, children could discuss what occurred in their daily lives during the COVID-19 pandemic.

Policy proposals: The children were able to recognise violations of their rights under each article and articulate the policy proposals needed to advance those rights.

Introduction

Well written and informative.

Minor editing:

P4 L15 incomplete sentence

→Regardless of these differences, school closures, staggered attendance, and shortened school days were implemented.

L17 restricted

→restricted

L33: standards for children

→standards for children

P5 L6-9 There is no need to detail the book title etc – this is in the references. Rather summarise the key concepts the authors propose

→We deleted the last sentence, instead,]we added the following:

Furthermore, the Convention on the Rights of the Child transformed children from "objects of protection (passive beings)" to "subjects of rights (active beings)" who possess their own rights.

When designing research on children, we researchers must prioritise the protection of children's rights and practice and establish research "with children" or "by children."

Section 2.1
Please explain why only girls were involved in creating the CRC articles

→According to the GCDRD reports by Global Child, they chose the girls' team because the girls' school had achieved a UNICEF Gold Award as a Rights Respecting School, and, as such, the pupils were already engaged with a rights-respecting language and ethos. We don't want to write this in the text. However, there is no need to write this in the text.

Results
P8 L22 is not clear – perhaps split into2 sentences. What happened if children turned 18 during the study?

→We have split it into paragraphs. And we revised this sentence a little as follows:

The children's ages ranged from 9 to 17 years; however, in Tanzania, the GCRD was held in secondary schools, so no children under 13 were recruited. In Japan, there were three 18-year-old participants, including older siblings and high school senior groups. In Japan, there were three 18-year-old participants, including older siblings of other participants and a group of high school seniors aged 17 and 18.
Tables could be far more engaging and clearly presented eg Table 1 list the ages (perhaps in categories in a column on left).

→We revised Table S1 (Old Table1).
Table 3 insert dividing lines between countries.
Consider a Table of Policy Recommendations as they seem to get lost throughout this article.
→We added two tables in the discussion section.

3.2.3 Tanzania
The 'level seats' issue should be explained the first time it is presented.
'Dedicated desks' may not be clear for an international audience – does this refer to call-in centres/drop in centres?
→The wording in "Level seat" is for 'Discussion'; here, only the facts are stated.

'The problem that children were excluded from public transport violated children's rights.'

Discussion
Overall well written and interesting. Please add more on the children's policy recommendations and possible next steps. Also please reflect more on how the unique experiences of children from different countries comes through in their perceptions. This is a very interesting aspect that is not explored.

→We added several sentences to each country. And we added two new tables for common violations, political proposals, and individual ones.
My suggestion is that '4.1 Current situation of the CRC' should be moved to the Introduction and properly integrated there. It disturbs the flow of the reader where it currently sits.

→We moved these sentences to the introduction section.

1.1. Current situation of the Convention on the Rights of the Child:

Japan became the 158th of 196 countries to ratify the CRC in April 1994. The prohibition of corporal punishment was codified in law through amendments to the Child Welfare Act and other relevant laws, and it came into effect in April 2020. The UN Committee on the Rights of the Child emphasised the need to allocate sufficient public budgets to improve the high child poverty rate, to enact comprehensive anti-discrimination laws, to recognise joint custody of children, and to ensure that no child is subject to trial in adult criminal courts. Because the age of criminal responsibility was lowered from 16 to 14 in 2000. Government surveys have revealed that many children are taking on household and family care roles during the pandemic, and that some of this care by young carers constitutes child labour.

Sweden was the first country in the world to legally prohibit corporal punishment of children in 1979. It was one of the original signatories to the CRC, which came into force in 1990. It established a Children's Ombudsman in 1993 and, in 2020, became the first country to incorporate the CRC into domestic law. However, challenges remain in implementing these principles in practice. Sahlberg et al. noted that children's medical care must explicitly take their rights into account. Furthermore, the 2023 Concluding Observations of the UN Committee on the Rights of the Child recommend strengthening children's participation in decision-making at the local level and ensuring equitable provision of public education for all children from socio-economically disadvantaged families, including refugees.

Tanzania ratified the CRC in 1991 but has not yet enacted legislation prohibiting corporal punishment. In its second periodic report in 2006, Tanzania was recommended to enact a Children's Act. This recommendation was followed in 2009 with the enactment of the Children's Act, formally titled the Law of the Child. In 2011, Human Rights Council of the UN pointed out several areas for improvement including concerns about the minimum marriage age of 15 for girls, the discrimination against children in socioeconomical difficulties and street children, problems with the functioning of juvenile councils, the lack of a provision prohibiting corporal punishment, the continued practice of female genital mutilation and the education law, which makes teenage pregnancy school dropouts.
'Strengths and limitations': please elaborate on any difficulties encountered with children with disabilities and what adaptations were made for them.

→We added a sentence.

'In such cases, we intervened by selecting the articles under discussion to be more specific ones, such as Article 31, which concerns the right to play and cultural activities, or by having a facilitator orient the discussion.'
P15 L13 It is not clear what is meant by the 'age restrictions in Tanzania"
→We added a sentence.

'Because the GCRD was implemented in junior and high schools, those under 12 years old were excluded. There were restrictions on age-range Tanzania.'

Conclusions
As before, what policy recommendations were the most significant that we need to listen to? How did children's unique country experiences during the pandemic affect their perception and recommendations? Why is this important ie it may be that we face future pandemics.
→We added a sentence.
'It has also confirmed the importance of our society listening to children's opinions, whether regarding future pandemics, existing conflicts, or climate crises.'

Reviewer: 2
Comments to the Author
Overall impression:

This article introduces a novel and important global topic for readers: the Global Child Rights Dialogue (GCRD) and 'Research with Children' approach. Moreover, the authors adopt GCRD in three countries in Africa, Asia and Europe. Therefore, the contents are worth publishing although it needs several major revisions. Especially, the whole structure of this manuscript seems not well organized (each content of **Background**, **Results** and **Discussion** is mixed up together) so changing the configuration of structure is recommended.
→We rewrote the text according to the reviewers' comments.

Comments for each section:
1. 'Policy Recommendations' in **Title** is a keyword of this article. However, it is difficult to find the policy recommendations in **Result** section although there are several sentences on **Figure 2,3,4**. Which parts are the policy recommendation? (one table will be needed).
→We revised the text's structure and added or revised sentences for better understanding.

Key Messages:
2. The whole writing style should be considered carefully again (Please refer to the published articles of BMJPO and following their writing structure. All sentences are written in the same way). Especially, **What this study adds**' part is written in distinctive styles, so it is difficult what they mean.

→Within the constraints of the text, we rewrote it as follows:

'Violations of children's rights: Through the same GCRD process, children could discuss what occurred in their daily lives during the COVID-19 pandemic.

Policy proposals: The children were able to recognise violations of their rights under each article and articulate the policy proposals needed to advance those rights.

Introduction
3. As 1-2, the basic information on children in Japan, Sweden and Tanzania should be explained (e.g., geographic characteristics, total fertility rate, school enrollment rate, rate of migrants…). In addition, the reason these three countries were selected should be explained.

→Based on your feedback, we have added a sub-chapter and tables.

1.3. Japan, Sweden and Tanzania

The selection of Japan, Sweden, and Tanzania was based on the first author's research connections (opportunity sampling). Basic Socio-economic data for these three countries, including children, sourced from the World Bank and OECD Data, are shown in Table 1.

Table 1 Indicators and data from each country

Country	Population 2023*		Fertility rate* 2023	GDP per capita* 2023	Gini index* (0-100)	Foreign population** 2024 (%)
	Total	Ages 0-14 %				
Japan	124,517,000	12	1.2	33,836	32.3 (2020)	3,400,000 (2.7%)***
Sweden	10,537,000	17	1.4	54,950	29.3 (2023)	2,200,000 (20.5%)****
Tanzania	66,618,000	43	4.6	1,224	40.5 (2018)	N.D.

* World Bank Data	** OECD Data	***Foreign population	****Foreign-born population

Methods

4. P5L19-20: study period'
What does it mean? (by the end of all interviews??)

→We rewrote 'The GCRD implemented period'.

5. P524-25: 'spanning from Ishinomaki City… in the west'
This part cannot be understood for foreigners because of too detail. So, this part maybe no need).

→It has significant meaning to involve the great earthquake area, so we keep this sentence.

6. P5L36: '**Patient and Public Involvement**':
What does it mean??

→This is necessary to submit to this journal.

7. P5L51: approval'
How was it obtained? Orally?

→Most requests were made by phone, and in some cases, we visited in person. No written agreements were exchanged at this stage. It is not necessary to write the details.

8. P6L10-31:
The process can be described as one paragraph. In the process **iv)**, the role of children should be explained, such as 'one volunteer for presentation'.
→We added "children's" to indicate that the children were the main. Depending on the group, one representative would explain, or everyone would take turns explaining.

'vi) The contents of the GCRD for each article and the policy proposals that emerged from it were presented by the children's group.'

9. P6L512: transcribed verbatim'
Did you use any recording tool? If so, it should be explained.
→If even one of the children objected to the recording, it wouldn't be done, so there were times when it was possible and times when it wasn't.

So, we rewrote 'All GCRD' into 'All GCRD recorded by the facilitators'.

10. P7L21-30:
In this part, it is difficult to understand why six group occurrences were adopted in Sweden and Tanzania after six groups emerged in Japan's GCRD. Please explain the process in more detail.

→We already explained the word-selection methods of the other two countries. So, we didn't add anything.

11. P7L37:
Are the meanings/definitions of 'policy proposals', 'policy demand' and 'policy recommendation' the same or not? Please use these key words with a clear purpose.

→Thank you very much for your valuable feedback. We had been using it without much thought. We have decided to use "proposals" as the standard. We have also corrected the article title, the titles of the table and other documents accordingly.

12. P7L33: sentences that briefly outline the messages within that group'
What is the relationship between this phrase and the 'policy recommendations?
→Both problems and policy proposals are shown based on the frequency of word occurrences. We added tables to make it easier to understand in the discussion section.

Results

13. **Table1**:

It is difficult to see tables because 1) the characters are too small, and 2) each item and numbers are not coordinated so we cannot understand the meaning of this table. Please remake it.

→We revised Table S1 (Old Table1).

14. **Table2 and Table3**:

The character of each item is too small.

→We also revised Table 2 and 3 as Table S2 and S3.

15. P10L19-20: 'The following…'

This sentence is no need.

→We deleted this sentence.

16. P11L40: Level seats :

These words appear suddenly so it needs some explanation or should be expressed with "". The explanation appeared on P14L44 for the first time.

→The wording in "Level seat" is for 'Discussion'; here, only the facts are stated.

'The problem that children were excluded from public transport violated children's rights.'

Discussion

17. At the first paragraph of Discussion, 1) strengths of this study, and 2) statement of main (two or three) discission points should be described.

→We added the new texts as the first paragraph.

'The policy proposals concluded by the children's discussions in the GCRD have been quantitatively analysed using the KH Coder method in the Results chapter. In this Discussion chapter, we aim to clarify the "Pearls of wisdom" that the children demonstrated through the GCRD, which surpass the thinking of adults, and the problem-solving proposals that could only be presented from children's perspectives, while focusing on r proposals based on the specific circumstances of each country and proposals for the protection of universal rights, and discuss them deeply with references.'

18. P11-12: 4.1 **Current situation of the CRC**

This paragraph is not discussion but basic information so it is better to move to **Introduction** section.

→We moved it to the introduction section.

19. P12-13: **4.2.1-4.2.3**

These discussions for three countries seem to be nearly results. It is not clear how the authors extract these contents from **Result** section. I apology repeating but where are policy recommendations which we find easily? And are they the same as policy proposals/policy demands/sentences that briefly outline the messages within that group' ? These parts make the reader confused.

→We added the new paragraph at the start of the discussion.

And also, we added two tables to show common child rights violations and policy proposals.

20. P14L54: Missing **4.3**

As one section (maybe 4.3?) in **Discussion**, it is better to put the reflection of comparison for policy recommendation among three countries, in a summary style. Such multi-countries evaluation will show the worth conducting the study in three countries.

→We added a new Table 2 for it.
21. P14L55: **Strengths**
To what extent this study is brand-new, innovative and unique should be underlined first. It means that it is important to mention how much the research with children- approach and GCRD are popular in the world when policy recommendations are formulated on CRC.
→We added two sentences to show the strengths.

'The strength of this study is embodying the concept of "research with children. By clearly explaining what GCRD is and which CRC article to discuss, children were able to proactively analyse the current state of their rights and make policy recommendations for improvement. This was possible despite weaknesses such as younger age or developmental disabilities. Children themselves took the lead in the GCRD project, discussed infringements of their rights due to COVID-19, and made policy proposals to address these issues. Therefore, this study is groundbreaking in that it clearly demonstrates the necessity of empowering and even enabling children to be active participants in advancing the articles of the CRC.'

Acknowledgements
22. It is written in heart-warning style but not formal. No need 'Thank you, XX.'
→We revised the acknowledgement in a much simpler way.

Reviewer: 1
If you have selected 'Yes' above, please provide details of any competing interests.: N/A
There is no COI.
Reviewer: 2
If you have selected 'Yes' above, please provide details of any competing interests.: Not applicable

There is no COI.
